# Should all patients with psoriasis receive statins? Analysis according to different strategies^☆^^☆☆^

**DOI:** 10.1016/j.abd.2019.03.001

**Published:** 2019-10-24

**Authors:** Walter Masson, Martín Lobo, Graciela Molinero, Emiliano Rossi

**Affiliations:** a Cardiology Service, Hospital Italiano de Buenos Aires, Buenos Aires, Argentina; b Council of Epidemiology and Cardiovascular Prevention, Sociedad Argentina de Cardiología, Buenos Aires, Argentina

**Keywords:** Hydroxymethylglutaryl-CoA reductase inhibitors, Lipids, Psoriasis

## Abstract

**Background:**

Different strategies have been proposed for the cardiovascular risk management of patients with psoriasis.

**Objective:**

To estimate the cardiovascular risk and evaluate two cardiovascular prevention strategies in patients with psoriasis, analyzing which proportion of patients would be candidates to receive statin therapy.

**Methods:**

A retrospective cohort was selected from a secondary database. All patients >18 years with psoriasis without cardiovascular disease or lipid-lowering treatment were included. The atherosclerotic cardiovascular disease calculator (2018 American College of Cardiology/American Heart Association guidelines) and the Systematic Coronary Risk Evaluation risk calculator (2016 European Society of Cardiology/European Society of Atherosclerosis guidelines) were calculated. The SCORE risk value was adjusted by a multiplication factor of 1.5. The recommendations for the indication of statins suggested by both guidelines were analyzed.

**Results:**

A total of 892 patients (mean age 59.9 ± 16.5 years, 54.5% women) were included. The median atherosclerotic cardiovascular disease calculator and Systematic Coronary Risk Evaluation values were 13.4% (IQR 6.1–27.0%) and 1.9% (IQR 0.4–5.2), respectively. According to the atherosclerotic cardiovascular disease calculator, 20.1%, 11.0%, 32.9%, and 36.4% of the population was classified at low, borderline, moderate, or high risk. Applying the Systematic Coronary Risk Evaluation, 26.5%, 42.9%, 20.8%, and 9.8% of patients were stratified as having low, moderate, high, or very high risk, respectively. The proportion of subjects with statin indication was similar using both strategies: 60.1% and 60.9% for the 2018 American College of Cardiology/American Heart Association and 2016 European Society of Cardiology/European Society of Atherosclerosis guidelines, respectively.

**Study limitations:**

This was a secondary database study. Data on the severity of psoriasis and pharmacological treatments were not included in the analysis.

**Conclusion:**

This population with psoriasis was mostly classified at moderate–high risk and the statin therapy indication was similar when applying the two strategies evaluated.

## Introduction

Chronic inflammatory disorders, including psoriasis, are characterized by enhanced atherosclerosis and consequently higher cardiovascular morbidity and mortality rates compared with the general population.^1^, ^2^, ^3^, ^4^

Therefore, particular attention should be paid to conventional cardiovascular risk factor treatment, including dyslipidemia, in these patients. Statins are effective in reducing disease activity, and in *post hoc* analysis of randomized clinical trials, statins improved lipid levels and cardiovascular outcomes in patients with and without psoriasis, supporting statin use in patients with psoriasis.^5^ However, there is no certain indication for the use of lipid-lowering therapy only on the basis of the presence of the disease.

The traditional risk scales used to estimate cardiovascular risk have great limitations; due to the fact that they were not developed specifically for psoriasis, they have a tendency to underestimate the risk.^6^, ^7^ The decrease in the indication of interventions in cardiovascular prevention, such as statins, may be the result of this deficient evaluation.

Two strategies have been proposed for the cardiovascular risk management of these patients. The first, recommended by the European Society of Cardiology (ESC), the European Society of Atherosclerosis (EAS), and the European League Against Rheumatism (EULAR), is to adjust the risk calculated by a multiplying factor (1.5×) and follow the recommendations for statin therapy of the general population.^8^, ^9^

The second strategy places psoriasis as a clinical situation that increases cardiovascular risk and consequently, it favors the indication of statins at least in subjects with intermediate risk. In this case, no adjustment factor is suggested. This strategy is recommended by the new American College of Cardiology/American Heart Association (ACC/AHA) guidelines for cholesterol management introduced at the end of 2018.^10^

Taking into account the previously mentioned considerations, the objectives of this study were as follows: (1) to estimate the cardiovascular risk measured by two different scores in patients with psoriasis without previous cardiovascular disease (CVD); (2) to evaluate two cardiovascular prevention strategies in patients with psoriasis, analyzing which proportion of patients would be candidates to receive statin therapy; (3) to establish the reasons that justify this indication.

## Methods

A retrospective cohort was selected from a secondary database (electronic medical records). The sample was obtained from a private health system constituted by two university hospitals and a network of 21 associated peripheral centers distributed in the city of Buenos Aires and in the province of Buenos Aires, Argentina. All patients older than 18 years with a diagnosis of psoriasis were included. Patients with previous CVD, chronic renal insufficiency, or concomitant lipid-lowering treatment were excluded.

The atherosclerotic cardiovascular disease (ASCVD) calculator used by the 2018 ACC/AHA guidelines for cholesterol management^10^ and the Systematic Coronary Risk Evaluation (SCORE) estimate the ten-year risk of ASCVD fatal events, corresponding to low-risk countries used by the 2016 ESC/EAS guidelines on CVD prevention in clinical practice,^8^ were calculated. The choice of SCORE, corresponding to low risk countries, was arbitrary, based on the fact that most of the Argentine immigrant population comes from those countries. The risk value calculated by the SCORE was adjusted by a multiplication factor of 1.5, following the latest European recommendations.^8^, ^9^ Following the recommendations of the mentioned guidelines, indications for statins with a level of recommendation I or IIa were selected for this analysis.

Applying the 2018 ACC/AHA guidelines, the following recommendations were taken into account for patients in primary prevention: (a) in patients 40–75 years of age with diabetes mellitus and LDL-C ≥70 mg/dL, start moderate-intensity statin therapy without estimating the ten-year ASCVD risk calculator; (b) in patients 20–75 years of age with an LDL-C level of 190 mg/dL or higher, high-intensity statin therapy is recommended without estimating the ten-year ASCVD risk calculator; (c) in adults 40–75 years of age without diabetes mellitus and ten-year risk ≥20%, start high-intensity statin therapy; (d) in adults 40–75 years of age without diabetes mellitus and ten-year risk of 7.5–19.9% (intermediate risk), risk-enhancing factors favor initiation of moderate-intensity statin therapy. Risk-enhancing factors include psoriasis.

Applying the 2016 ESC/EAS guidelines, the following recommendations were taken into account for patients in primary prevention: (a) in patients with diabetes >40 years of age, start moderate/high-intensity statin therapy without estimating SCORE risk calculator; (b) in patients with an LDL-C level of 190 mg/dL or higher, statin therapy is recommended; (c) in adults >40 years of age without evidence of CVD or diabetes with a calculated SCORE ≥1% and <5% for ten-year fatal CVD and LDL-C ≥100 mg/dL, start statin therapy; (d) in adults >40 years of age without evidence of CVD or diabetes with a calculated SCORE ≥5% and <10% for ten-year fatal CVD and LDL-C ≥70 mg/dL, start statin therapy; (e) in adults >40 years of age without evidence of CVD or diabetes with a calculated SCORE ≥10% for ten-year risk of fatal CVD, start statin therapy.

Continuous data between two groups were analyzed using Student's *t*-test if the variables were normally distributed or with the Wilcoxon–Mann–Whitney test otherwise. Categorical data analysis was performed using the chi-squared test. Continuous variables are given as mean ± standard deviation, while categorical variables are given as percentages. The agreement between both strategies in selecting patients with statin indication was analyzed, using the Fleiss kappa index. Mild or poor, acceptable or discrete, moderate, significant, or almost perfect agreement was defined if the kappa value was <0.20, between 0.21 and 0.40, 0.41 and 0.60, 0.61 and 0.80, or 0.81 and 1, respectively. The chi-squared test for homogeneity was performed to compare between kappa values. A value of *p* < 0.05 was considered statistically significant. STATA v. 13.0 and EPIDAT v. 3.1 software packages were used for statistical analysis.

The study was conducted in compliance with the recommendations for medical research contained in the Declaration of Helsinki, the Good Clinical Practice standards, and the applicable ethical regulations.

## Results

A total of 892 patients (mean age 59.9 ± 16.5 years, 54.5% women) were included in the study. The average body mass index was 28.2 ± 5.9 and the mean HDL-C, triglyceride, and total cholesterol values were 51.5 ± 14.5 mg/dL, 119.6 ± 70.5 mg/dL, and 198.3 ± 42.2 mg/dL, respectively. Importantly, 54.3% of patients were hypertensive and 24.8% were active smokers. The baseline characteristics of the population are described in Table 1.

**Table 1:** Characteristics of the population.

*Continuous variables, mean (SD)*	*n* = 892
Age, years	59.9 (16.5)
Systolic blood pressure, mmHg	127.5 (15.9)
Diastolic blood pressure, mmHg	77.9 (9.7)
Body mass index, kg/m^2^	28.2 (5.9)
Total cholesterol, mg/dL	198.3 (42.2)
LDL-C, mg/dL	123.6 (34.2)
HDL-C, mg/dL	51.5 (14.5)
Triglycerides, mg/dL	119.6 (70.5)
Non HDL-C, mg/dL	146.9 (40.5)
Blood glucose, mg/dL	100.8 (25.8)
HbA1c, % (patients with diabetes)	6.3 (1.0)
Creatinine, mg/dL	0.87 (0.22)

*Categorical variables (%)*	
Male gender	45.5
Hypertension	54.3
Smoking	24.8
Diabetes	13.9
Obesity	30.9
Psoriatic arthritis	7.0

The median 2018 ACC/AHA score and SCORE risk calculator values were 13.4% (IQR 6.1–27.0%) and 1.9% (IQR 0.4–5.2), respectively. According to the 2018 ACC/AHA score, 20.1%, 11.0%, 32.9%, and 36.4% of the population was classified at low, borderline, moderate, or high risk, respectively. Applying the SCORE risk calculator, 26.5%, 42.9%, 20.8%, and 9.8% of patients were stratified as having low, moderate, high, or very high risk, respectively.

**Figure 1:**
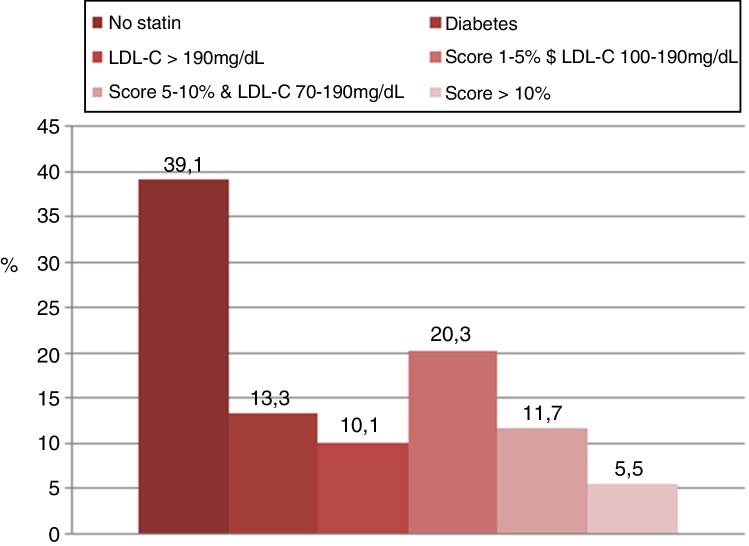
The reasons why statin therapy was indicated using the 2016 European Society of Cardiology/European Society of Atherosclerosis (ESC/EAS) guidelines.

**Figure 2:**
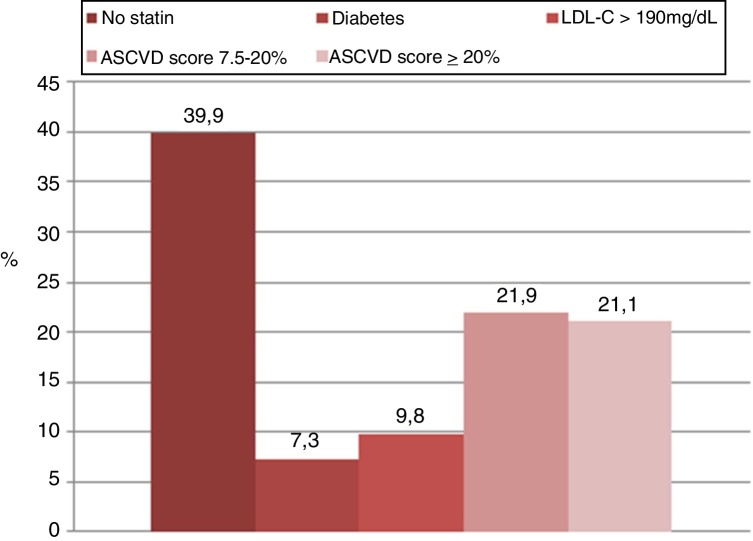
The reasons why statin therapy was indicated using 2018 American College of Cardiology/American Heart Association (ACC/AHA) guidelines.

Overall, the proportion of subjects with statin indication was similar using both strategies (*p* = 0.91). According to the 2018 ACC/AHA guidelines and based on the ASCVD calculator, the use of statins was recommended in 60.1% of cases. When the 2016 ESC/EAS guidelines were applied using the SCORE risk calculator, statins were recommended in 60.9% of cases. However, the concordance between both strategies in selecting patients with statin indication was moderate (*κ* = 0.46). The reasons why statin therapy was indicated using both strategies are shown in Figure 1, Figure 2.

The patients with statin therapy indication, by both guidelines, showed more cardiovascular risk factors and a higher prevalence of psoriatic arthritis than subjects without this pharmacological indication. Characteristics of the population according to the indication of statin therapy recommended by the 2018 ACC/AHA and 2016 ESC/EAS guidelines can be seen in Table 2.

**Table 2:** Characteristics of the population according to the indication of statin therapy recommended by the 2018 ACC/AHA and the 2016 ESC/EAS guidelines.

	2018 ACC/AHA guidelines		
	With statin-indication (*n* = 536)	Without statin-indication (*n* = 356)	*p*
*Continuous variables, mean (SD)*			
Age, years	63.7 (11.6)	54.3 (12.7)	<0.001
Systolic blood pressure, mmHg	131.4 (16.2)	121.7 (13.7)	<0.001
Diastolic blood pressure, mmHg	79.8 (9.7)	75.0 (9.1)	<0.001
Body mass index, kg/m^2^	28.9 (6.2)	27.1 (5.6)	0.002
Total cholesterol, mg/dL	202.1 (44.1)	192.7 (38.4)	0.001
LDL-C, mg/dL	128.1 (35.9)	117.3 (30.6)	<0.001
HDL-C, mg/dL	49.8 (13.8)	54.1 (15.1)	<0.001
Triglycerides, mg/dL	125.7 (67.9)	110.4 (73.4)	0.002
Non HDL-C, mg/dL	152.4 (42.1)	138.7 (36.3)	<0.001
Blood glucose, mg/dL	103.9 (29.4)	96.2 (18.2)	<0.001
Creatinine, mg/dL	0.91 (0.26)	0.88 (0.26)	0.362

*Categorical variables (%)*			
Male gender	57.5	27.5	<0.001
Hypertension	66.9	35.1	<0.001
Smoking	29.5	17.7	<0.001
Diabetes	14.9	12.4	0.278
Obesity	37.4	21.4	<0.001
Psoriatic arthritis	8.6	4.5	0.019

	2016 ESC/EAS guidelines		
	With statin-indication (*n* = 536)	Without statin-indication (*n* = 356)	*p*
*Continuous variables, mean (SD)*			
Age, years	67.4 (12.7)	47.9 (14.7)	<0.001
Systolic blood pressure, mmHg	130.7 (16.3)	122.5 (13.9)	<0.001
Diastolic blood pressure, mmHg	78.7 (9.6)	76.6 (9.7)	0.002
Body mass index, kg/m^2^	28.6 (6.1)	27.5 (5.8)	0.074
Total cholesterol, mg/dL	203.2 (42.6)	190.5 (40.2)	0.001
LDL-C, mg/dL	129.4 (34.3)	115.5 (32.4)	<0.001
HDL-C, mg/dL	51.5 (14.9)	51.3 (13.9)	0.865
Triglycerides, mg/dL	124.2 (70.8)	112.2 (69.5)	0.014
Non HDL-C, mg/dL	151.7 (40.7)	139.2 (38.8)	<0.001
Blood glucose, mg/dL	105.4 (30.6)	93.5 (12.2)	<0.001
Creatinine, mg/dL	0.91 (0.27)	0.87 (0.64)	0.341

*Categorical variables (%)*			
Male gender	50.6	37.4	<0.001
Hypertension	66.2	35.1	<0.001
Smoking	26.7	21.6	0.08
Diabetes	21.9	1.4	<0.001
Obesity	34.3	25.4	<0.001
Psoriasic arthritis	7.9	5.4	0.196

In the analysis according to sex, men had a greater indication for statin therapy compared to women (75.9% *vs*. 46.9%, *p* < 0.001) according to the 2018 ACC/AHA guidelines. Similar findings were found when applying 2016 ESC/ESC guidelines (men 67.7% *vs*. women 55.1%, *p* < 0.001). The application of the 2018 ACC/AHA guidelines selected a higher proportion of men with statin indication in comparison with the 2016 ESC/ESC guidelines (75.9% *vs*. 67.7%, *p* = 0.01). Contrarily, the use of the 2016 ESC/ESC guidelines selected a higher proportion of women with statin indication in comparison with the 2018 ACC/AHA guidelines (55.1% *vs*. 46.9%, *p* = 0.005).

**Figure 3:**
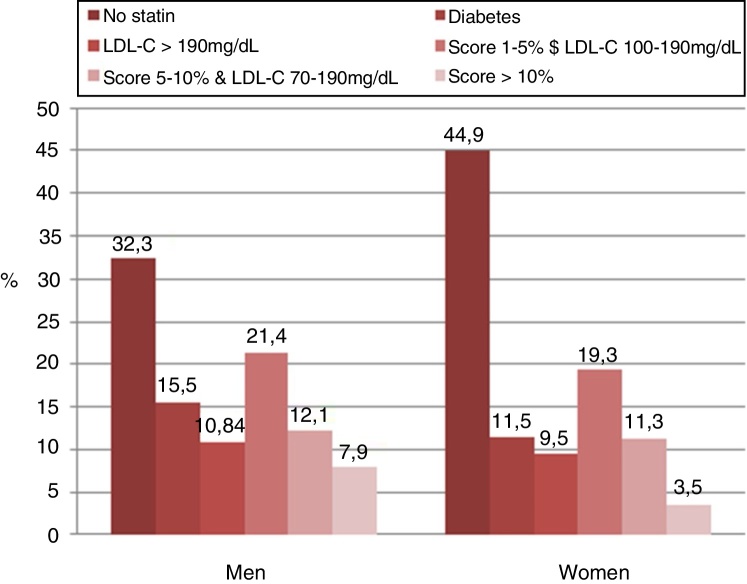
The reasons why statin therapy was indicated using the 2016 European Society of Cardiology/European Society of Atherosclerosis (ESC/EAS) guidelines according to sex.

**Figure 4:**
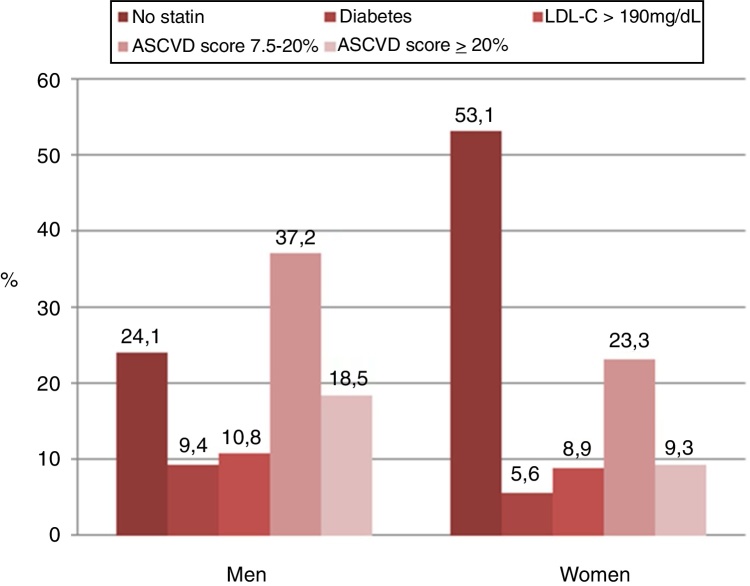
The reasons why statin therapy was indicated using the 2018 American College of Cardiology/American Heart Association (ACC/AHA) guidelines according to sex.

The concordance between two strategies in selecting patients with statin indication was moderate in both sexes (men: *κ* = 0.46, women: *κ* = 0.42; *p* = 0.29). The reasons why statin therapy was indicated using both strategies in the analysis according to sex showed in Figure 3, Figure 4.

## Discussion

Psoriasis is a chronic inflammatory skin disease associated with increased cardiovascular morbidity and mortality.^11^, ^12^ Several mechanisms have been proposed to explain the relationship between psoriasis and cardiovascular risk. Indeed, patients with psoriasis have an increased prevalence of classic cardiovascular risk factors, including obesity, hypertension, diabetes, dyslipidemia, metabolic syndrome, and nonalcoholic fatty liver disease.^13^, ^14^, ^15^, ^16^ However, psoriasis may provide an additional and independent cardiovascular risk factor, most likely because several cytokines (tumor necrosis factor-alpha, interferon, interleukin-17, interleukin-6) released by skin lesions can directly favor the development and progression of atherosclerosis.^17^ Likewise, the risk of CVDs is increased in chronic inflammatory disorders, with evidence that risk is associated with severity of inflammation.^18^

The SCORE risk calculator is recommended for CVD risk prediction in the general population by the ESC/EAS guidelines. However, CVD risk prediction models developed for the general population do not include non-traditional CVD risk factors. If these models are applied in patients with psoriasis, there is a possibility of underestimating the CVD risk.

In addition to appropriate cardiovascular risk management, some authors believe that the risk score models should be adapted for patients with psoriasis by introducing a multiplication factor that takes into account the presence of psoriasis.^9^

Other authors establish that psoriasis is a “risk enhancer” that can be used to favor initiation or intensification of statin therapy, particularly in stratified patients with intermediate risk. This strategy, based on the ASCVD calculator, is recommended by the new 2018 ACC/AHA guidelines for cholesterol management and does not contemplate any multiplication or adjustment factor.^10^

In the present study, when it was applied the 2018 ACC/AHA guidelines, the majority of patients were classified as intermediate or high risk. However, when it was applied the 2016 ECS/EAS guidelines, the majority of patients with psoriasis were stratified as intermediate risk. These finding coincides with a cross-sectional study of 234 patients with psoriasis that showed that the cardiovascular risk estimated by the Framingham score was on average 11.2% (intermediate risk).^19^ Similarly, a study that analyzed 395 patients with psoriasis showed that the proportion of patients at intermediate and high risk of suffering a major cardiovascular event in the next ten years was 30.5% and 11.4%, respectively, based on the Framingham risk score.^7^ Another study conducted in Brazil involving the assessment of 190 patients with psoriasis showed that 47% had moderate or high risk of fatal and non-fatal coronary events in ten years.^20^

The main finding of the study was that by using two different strategies of cardiovascular prevention for the management of patients with psoriasis, the proportion of eligible patients for statin therapy was similar (close to 60%). However, the concordance between both strategies in selecting patients with statin indication was moderate, indicating that individually, some subjects had different indications according to the guideline used.

These findings differ from another study that evaluated patients with rheumatoid arthritis. Tournadre et al. calculated the proportion of patients eligible for statins according to ESC guidelines, the Adult Treatment Panel III, and the ACC/AHA in a French cohort of statin-naïve rheumatoid arthritis patients at least 40 years of age. A marked discordance in risk assessment and cholesterol treatment was observed between the three sets of guidelines.^21^ The difference with the present work could be explained by the populations studied, plus the fact that a multiplication factor was used in this study.

Several publications showed that men more frequently receive an indication of statin therapy than women when analyzing the general population.^22^, ^23^ Regarding this topic, but analyzing only patients with psoriasis, the present study showed that the proportion of subjects with an indication for statin therapy was higher in men than women, regardless of the chosen strategy.

To determine whether a patient is a candidate for statin therapy, clinicians must first determine the patient's risk of having a future CVD event. However, clinicians’ ability to accurately identify a patient's true risk is imperfect, because the currently available risk estimation tools have been shown to underestimate the risk in patients with chronic inflammatory diseases.^6^, ^24^, ^25^ Consequently, the current methods of cardiovascular risk assessment in the course of chronic inflammatory diseases are a subject of considerable controversy.

In the present study, the main reason for the indication of statin treatment was the intermediate cardiovascular risk calculated with the scoring method. In addition, this finding was observed when using both strategies and in both sexes. These findings reinforce the importance of using tools for the stratification of cardiovascular risk, despite its limitations.

According to the results of this study, the authors believe that not all patients with psoriasis should receive statins. European and North American strategies, analyzed in this study, agree that patients in primary prevention with diabetes and/or a level of LDL-C >190 mg/dL should receive statins, whether they have psoriasis or not. The other group of patients, who are candidates for statins, should be defined according to the estimated cardiovascular risk. Europeans guidelines use SCORE and adjust the value by a correction factor. Americans guidelines use the ASCVD calculator and do not use an adjustment factor. Both strategies will recommend giving statins to patients at intermediate or high risk.

In synthesis, every psoriatic patient with a very high cholesterol value, diabetes, or an intermediate/high risk should receive statins. Taking into account that patients with psoriasis have a higher cardiovascular risk independently of the presence of conventional risk factors, it would be advisable to apply a correction factor to the cardiovascular risk score calculated as the Europeans recommendations do.

This study had some limitations. It was a secondary database study (electronic medical records); consequently, there could be information bias. Furthermore, the data on the severity of psoriasis and pharmacological treatments could not be reliably obtained retrospectively; therefore, this data could not be included in the analysis. Despite its limitations, this study represents a valuable contribution, as a large group of patients with psoriasis but without CVD was examined. Research for risk factors and proper risk stratification are rare in patients with psoriasis. Knowledge of the application of different strategies in cardiovascular prevention could favor the difficult task of estimating cardiovascular risk in this particular group of patients.

## Conclusion

This study's findings showed that not all patients with psoriasis should receive statins. The population with psoriasis without CVD was mostly classified at moderate–high risk, and the statin therapy indication was similar when applying the two strategies evaluated.

## Financial support

None declared.

## Author's contribution

Walter Masson: Statistical analysis; approval of the final version of the manuscript; conception and planning of the study; elaboration and writing of the manuscript; obtaining, analyzing and interpreting the data; critical review of the literature; critical review of the manuscript.

Martín Lobo: Statistical analysis; approval of the final version of the manuscript; obtaining, analyzing and interpreting the data.

Graciela Molinero: Approval of the final version of the manuscript; conception and planning of the study; elaboration and writing of the manuscript; critical review of the manuscript.

Emiliano Rossi: Statistical analysis; approval of the final version of the manuscript; elaboration and writing of the manuscript; obtaining, analyzing and interpreting the data; critical review of the literature.

## Conflicts of interest

None declared.

## References

[1] Gelfand J.M., Neimann A.L., Shin D.B., Wang X., Margolis D.J., Troxel A.B. (2006). Risk of myocardial infarction in patients with psoriasis. JAMA.

[2] Lauper K., Courvoisier D.S., Chevallier P., Finckh A., Gabay C. (2018). Incidence and prevalence of major adverse cardiovascular events in rheumatoid arthritis, psoriatic arthritis, and axial spondyloarthritis. Arthritis Care Res (Hoboken).

[3] Masson W., Rossi E., Galimberti M.L., Krauss J., Navarro Estrada J., Galimberti R. (2017). Mortality in patients with psoriasis. A retrospective cohort study. Med Clin (Barc).

[4] Mehta N.N., Azfar R.S., Shin D.B., Neimann A.L., Troxel A.B., Gelfand J.M. (2010). Patients with severe psoriasis are at increased risk of cardiovascular mortality: cohort study using the General Practice Research Database. Eur Heart J.

[5] Ports W.C., Fayyad R., DeMicco D.A., Laskey R., Wolk R. (2017). Effectiveness of lipid-lowering statin therapy in patients with and without psoriasis. Clin Drug Investig.

[6] Eder L., Chandran V., Gladman D.D. (2014). The Framingham Risk Score underestimates the extent of subclinical atherosclerosis in patients with psoriatic disease. Ann Rheum Dis.

[7] Fernández-Torres R., Pita-Fernández S., Fonseca E. (2013). Psoriasis and cardiovascular risk. Assessment by different cardiovascular risk scores. J Eur Acad Dermatol Venereol.

[8] Piepoli M.F., Hoes A.W., Agewall S., Albus C., Brotons C., Catapano A.L. (2016). 2016 European Guidelines on cardiovascular disease prevention in clinical practice: the Sixth Joint Task Force of the European Society of Cardiology and Other Societies on Cardiovascular Disease Prevention in Clinical Practice (constituted by representatives of 10 societies and by invited experts) developed with the special contribution of the European Association for Cardiovascular Prevention & Rehabilitation (EACPR). Atherosclerosis.

[9] Agca R., Heslinga S.C., Rollefstad S., Heslinga M., McInnes I.B., Peters M.J.L. (2017). EULAR recommendations for cardiovascular disease risk management in patients with rheumatoid arthritis and other forms of inflammatory joint disorders: 2015/2016 update. Ann Rheum Dis.

[10] Grundy S.M., Stone N.J., Bailey A.L., Beam C., Birtcher K.K., Blumenthal R.S. (2018). 2018 AHA/ACC/AACVPR/AAPA/ABC/ACPM/ADA/AGS/APhA/ASPC/NLA/PCNA Guideline on the Management of Blood Cholesterol: a report of the American College of Cardiology/American Heart Association Task Force on Clinical Practice Guidelines. J Am Coll Cardiol.

[11] Augustin M., Vietri J., Tian H., Gilloteau I. (2017). Incremental burden of cardiovascular comorbidity and psoriatic arthritis among adults with moderate-to-severe psoriasis in five European countries. J Eur Acad Dermatol Venereol.

[12] Fernández-Gutiérrez B., Perrotti P.P., Gisbert J.P., Domènech E., Fernández-Nebro A., Cañete J.D. (2017). Cardiovascular disease in immune-mediated inflammatory diseases: a cross-sectional analysis of 6 cohorts. Medicine (Baltimore).

[13] Gottlieb A.B., Dann F. (2009). Comorbidities in patients with psoriasis. Am J Med.

[14] Gisondi P., Targher G., Zoppini G., Girolomoni G. (2009). Non-alcoholic fatty liver disease in patients with chronic plaque psoriasis. J Hepatol.

[15] Masson W., Galimberti M.L., Anselmi C.L., Cagide A., Galimberti R.L. (2013). Coronary artery disease in patients with psoriasis. Medicina (B Aires).

[16] Adışen E., Uzun S., Erduran F., Gürer M.A. (2018). Prevalence of smoking, alcohol consumption and metabolic syndrome in patients with psoriasis. An Bras Dermatol.

[17] Nestle F.O., Kaplan D.H., Barker J. (2009). Psoriasis. N Engl J Med.

[18] Dregan A., Charlton J., Chowienczyk P., Gulliford M.C. (2014). Chronic inflammatory disorders and risk of type 2 diabetes mellitus, coronary heart disease, and stroke: a population-based cohort study. Circulation.

[19] Gisondi P., Farina S., Giordano M.V., Girolomoni G. (2010). Usefulness of the Framingham risk score in patients with chronic psoriasis. Am J Cardiol.

[20] Baeta I.G., Bittencourt F.V., Gontijo B., Goulart E.M. (2014). Comorbidities and cardiovascular risk factors in patients with psoriasis. An Bras Dermatol.

[21] Tournadre A., Tatar Z., Pereira B., Chevreau M., Gossec L., Gaudin P. (2015). Application of the European Society of Cardiology, Adult Treatment Panel III and American College of Cardiology/American Heart Association guidelines for cardiovascular risk management in a French cohort of rheumatoid arthritis. Int J Cardiol.

[22] Masson W., Lobo M., Huerín M., Molinero G., Manente D., Pángaro M. (2014). Use of different scores for cardiovascular risk stratification in primary prevention and their implications in statin indication. Rev Argent Cardiol.

[23] Wallach-Kildemoes H., Stovring H., Holme Hansen E., Howse K., Pétursson H. (2016). Statin prescribing according to gender, age and indication: what about the benefit-risk balance?. J Eval Clin Pract.

[24] Boulos D., Koelmeyer R.L., Morand E.F., Hoi A.Y. (2017). Cardiovascular risk profiles in a lupus cohort: what do different calculators tell us?. Lupus Sci Med.

[25] Bonek K., Głuszko P. (2016). Cardiovascular risk assessment in rheumatoid arthritis – controversies and the new approach. Reumatologia.

